# Debates in Pancreatic Beta Cell Biology: Proliferation Versus Progenitor Differentiation and Transdifferentiation in Restoring β Cell Mass

**DOI:** 10.3389/fendo.2021.722250

**Published:** 2021-08-06

**Authors:** Erick Spears, Ioannis Serafimidis, Alvin C. Powers, Anthony Gavalas

**Affiliations:** ^1^Division of Diabetes, Endocrinology and Metabolism, Department of Medicine, Vanderbilt University Medical Center, Nashville, TN, United States; ^2^Center of Basic Research, Biomedical Research Foundation of the Academy of Athens, Athens, Greece; ^3^Department of Molecular Physiology and Biophysics, Vanderbilt University School of Medicine, Nashville, TN, United States; ^4^VA Tennessee Valley Healthcare System, Nashville, TN, United States; ^5^Paul Langerhans Institute Dresden (PLID) of Helmholtz Center Munich at the University Clinic Carl Gustav Carus of TU Dresden, Helmholtz Zentrum München, German Research Center for Environmental Health, Neuherberg, Germany; ^6^German Centre for Diabetes Research (DZD), Neuherberg, Germany

**Keywords:** β cells, β cell proliferation, differentiation, transdifferentiation, acinar cells, duct cells, centroacinar cells

## Abstract

In all forms of diabetes, β cell mass or function is reduced and therefore the capacity of the pancreatic cells for regeneration or replenishment is a critical need. Diverse lines of research have shown the capacity of endocrine as well as acinar, ductal and centroacinar cells to generate new β cells. Several experimental approaches using injury models, pharmacological or genetic interventions, isolation and *in vitro* expansion of putative progenitors followed by transplantations or a combination thereof have suggested several pathways for β cell neogenesis or regeneration. The experimental results have also generated controversy related to the limitations and interpretation of the experimental approaches and ultimately their physiological relevance, particularly when considering differences between mouse, the primary animal model, and human. As a result, consensus is lacking regarding the relative importance of islet cell proliferation or progenitor differentiation and transdifferentiation of other pancreatic cell types in generating new β cells. In this review we summarize and evaluate recent experimental approaches and findings related to islet regeneration and address their relevance and potential clinical application in the fight against diabetes.

## Introduction

Insulin-producing β cells are critical for maintaining systemic glucose homeostasis. In both type 1 diabetes (T1D) and type 2 diabetes (T2D), reduced and/or inadequate β cell mass leads to insufficient insulin secretion and hyperglycemia. T1D is the result of autoimmune destruction of β cells. At clinical T1D onset, there are still remaining β cells, but the β cell mass declines further with increasing T1D duration. However, many individuals with long-standing T1D continue to secrete some insulin, indicating the persistence of some β cells. In T2D, the combination of insulin resistance due to obesity and impaired functional β cell mass leads to hyperglycemia. With increasing duration of T2D, β cell mass and function progressively declines. Thus, in both forms of diabetes, there is a great need to restore or increase β cell mass (1). In this review, we compare and contrast two primary mechanisms by which β cell mass can be increased: 1) differentiation of progenitor/stem cells or transdifferentiation of other pancreatic cell types into new β cells; and 2) stimulation of proliferation of remaining β cells. These two approaches are discussed with respect to their physiological relevance with the Pros and Cons of each discussed below and in **Table 1**. While presented as two distinct processes, in reality, both may play a critical role in restoring β cell mass. Prior to discussing these mechanisms, we first review important experimental considerations.

**Table 1:** Pros and Cons of β cell proliferation versus neogenesis through progenitor differentiation or transdifferentiation.

Mode of β Cell Regeneration		Pros	Cons
Mechanism	Original Cell Type		
**Proliferation**	β cells	• Most direct route to β cell regeneration • Many mitogenic pathways defined • Some success with combined pathway approach	• Low rate of adult β cell proliferation • Mitogenic pathways identified using Injury models may not be applicable in disease • Reliance on residual β cell mass in diseased pancreas
**Differentiation**	Dedifferentiated β cells	• Dedifferentiated cells generated as a result of diabetes • Reversal of autoimmunity or restoration of normoglycemia can induce differentiation • Differentiation can be combined with α cell transdifferentiation	• The number of dedifferentiated cells is controversial • Advanced diabetes may exhaust this pool
	Endocrine progenitors	• ϵ cells contribute to α and PP cells • Evidence for endocrine progenitors in mouse islets	• Conversion of ϵ cells to β cells is rare • No evidence of endocrine progenitors in human islets
	Centroacinar cells	• Developmental origins indicate potential for maintaining progenitor status • Proliferate in response to β cell destruction • Lineage tracing evidence for β cell neogenesis in homeostasis	• Heterogeneous cell population • Lack of suitable markers for centroacinar cell isolation • No lineage tracing evidence for β cell neogenesis in diabetic conditions
**Transdifferentiation**	α cells	• Well defined mechanism for stimulating α cell proliferation • α-to-β cell transdifferentiation has been observed in mice • Mouse genetic models have shown increased β cell mass *via* this mechanism	• Evidence of α to β cell transdifferentiation in humans is inconclusive • Lack of lineage tracing data in human islets • The presence of bi-hormonal and “virgin” β cells may mean that differentiation remains incomplete
	Acinar cells	• Most abundant pancreatic cell population • Acinar compartment contains actively proliferating subpopulations • Acinar cells have potential for transdifferentiation	• Only a small fraction of acinar cells can undergo transdifferentiation • Conversion not detected in uninjured pancreas • Actively proliferating acinar subpopulations have not shown capacity for β cell differentiation • Greatest potential for transdifferentiation into duct but not endocrine cells
	Duct cells	• Diverse cell populations that can be mobilized in response to different stimuli • Rat injury models indicate differentiation of β cells from regenerating ducts • Pancreatectomy results in reformation of complete pancreatic lobes from ducts • Rodent and human duct cells have potential for β cell transdifferentiation in *vitro*	• Controversy as to whether β cells arise by neogenesis from duct cells or proliferation in injury models • Duct regeneration is stimulated by immune responses, but this may negatively impact β cells • *In vivo* evidence for human duct-to-β cell transdifferentiation is lacking

First, proliferation and differentiation are important processes in establishing and maintaining baseline β cell mass. However, for diabetes-relevant studies, it is desirable to begin with reduced β cell mass that is subsequently restored since what determines restoration may differ from the determinants of baseline β cell mass. Several experimental approaches can be used to reduce β cell mass and the approach taken may influence the experimental outcome of β cell restoration. For example, chemical ablation of β cells with toxins such as streptozotocin or alloxan may impact not only the β cell, but also the progenitor cells responsible for restoration of β cell mass. In contrast, a genetic approach, with deletion of a gene leading to reduced β cell mass, is the product of irreversible genetic alterations that may not be truly representative of the genetic landscape of the natural disease state.

Second, while studying human tissues is important, there are considerable limitations and challenges. Although major strides have been made in the acquisition, processing, and manipulation of human pancreatic islets in recent years, human islets available for research are still relatively scarce. Among tissues that are procured for research, there is wide variability in physiologic responses due to cross-sectional genetic variability in the human population. Efforts to address this scarcity and genetic variability have employed directed differentiation of human pluripotent stem cells into islet cells in culture. These techniques have met with some success, but this is still a relatively new area of research and suffers from the limitations seen in most static culture models.

Third, mice have many strengths but some experimental limitations. Insulin promoter-driven Cre recombinase or fluorescence reporter lines, for β cell targeted gene knockout or β cell-specific reporter expression, have shown variations in β cell specificity with unwanted off-target effects. Furthermore, while mouse β cells show greater regenerative potential than human β cells, their regeneration is still difficult to stimulate. Many of the techniques used to assess β cell regeneration in mice, such as high fat diet or partial pancreatectomy, represent extreme stresses that may not be physiologically relevant to human islets, even those under the physiological stress that result in β cell loss during diabetes.

Fourth, there are profound differences between human and mouse islets that must be considered when relating islet studies performed in mice to humans. Mouse islets have a different architecture than human islets, having α cells on the outside of the islet surrounding a core of β cells. In contrast, human islets have interspersed α and β cells, implying differences in intercellular contacts. Mouse islets have a much higher β:α cell ratio (approximately 7:1) than human islets, in which the β:α ratio is closer to 2:1. Mouse islet cells have shown greater plasticity with respect to proliferation, dedifferentiation and transdifferentiation than do human islets. As discussed below, many of the individual mitogenic pathways in β cells were identified in mouse experiments. Unfortunately, the targeting of individual pathways in human β cells was not sufficient to stimulate significant proliferation as it was in mice. Only when combinatorial approaches were employed did human β cells achieve proliferation to a level that could potentially restore β cell mass in a diabetic setting. All of these differences, among others, indicate that caution is required when translating findings from mice to humans.

Taken together, these experimental considerations represent challenges that must be overcome when assessing the mechanisms of adult β cell regeneration. A consideration of the developmental origin of β cells and their lineage relationship with the other pancreatic cell types is also important as it can illuminate the molecular mechanisms of β cell proliferation and regeneration in the adult. All pancreatic cell types are derived from the same pancreas progenitor population that emerges at the posterior foregut region of the developing embryo. Pancreas progenitors are defined by the expression of the transcription factors PDX1, SOX9 and PTF1A and, as development proceeds, they form a branched epithelium. Acinar progenitors are gradually confined at the tips of this epithelium while trunk cells become bipotent endocrine/duct progenitors. All endocrine progenitors arise in the trunk as NGN3^+^ cells and migrate into the mesenchyme to form the islets that contain insulin producing β-cells (2, 3). Progenitor differentiation gives way to proliferation which increases β cell mass during late fetal and early neonatal development. The capacity for β cells to proliferate decreases with age. Fetal and neonatal β cells proliferate at the highest rates while adult β cells proliferation, under normal physiological conditions, is very low meaning that β cell mass is fixed in early adolescence.

Taking into account these experimental considerations, the goal of this review will be to detail the current understanding of the molecular and cellular mechanisms involved in β cell regeneration. Previous attempts at β cell regenerative therapies have been extensively reviewed recently (1, 4, 5). Our goal is not to repeat this information, but instead, we hope the reader will gain a comprehensive view of the potential for β cell regeneration and a novel understanding of the underlying molecular and cellular mechanisms. This discussion is accompanied by our assessment of the pros and cons of targeting these different mechanisms of β cell regeneration in hopes of inspiring new and innovative ideas about potential β cell regeneration therapies.

The challenge for adult β cell regeneration lies in reverting pancreatic cell types to an earlier developmental state that will allow them to regain their proliferative capacity and differentiate to fully functional β cells. Resident progenitor or stem cells would be an additional route for the restoration of functional β cell mass.

## Endocrine Cell Proliferation

Upon differentiation from progenitors during fetal pancreas development, early β cells expand through proliferation (6). While necessary to increase β cell mass in the fetal and neonatal pancreas, β cell proliferation declines rapidly into early adolescence and is essentially nonexistent in adulthood. The lack of proliferative capacity in adult β cells becomes problematic in individuals who develop diabetes because both T1D and T2D are associated with loss of β cell mass at some point during their progression. For this reason, major emphasis in diabetes research has been placed on understanding β cell proliferation during development and stimulating β cell proliferation in adults.

### Fetal and Neonatal β Cell Proliferation

Early studies in the mouse demonstrated a critical role for β cell proliferation in the establishment of adult β cell mass (6, 7). This indicates that it is the systemic environment in young animals that allows for the expansion of β cell mass through proliferation. The proliferative capacity observed in these early β cells is lost in adults due to decreases in stimulatory humoral factors (8), increased cyclin-dependent kinase inhibitor, p16^INK4A^, expression (9) and decreased responsiveness to PDGF signaling due to decreased expression of the PDGF receptor (10). The upregulation of cell cycle inhibitors and loss of growth factor signaling clamps down the intrinsic proliferative capacity of β cells in adults. Interestingly, parabiosis experiments with young and old mice have shown that the humoral factors which stimulate β cell proliferation in young animals may return adults β cells to a partially proliferation competent state (11). The proliferative capacity of β cells seems to be in direct contrast to their functional capacity. The proliferative fetal and neonatal β cells lack a robust glucose-stimulated insulin response. As these cells mature functionally and become more glucose-responsive, they lose the ability to proliferate (12). The loss of proliferative capacity in adult β cells may be necessary for this functional maturation and is the product of a shift in the nutritional environment after birth (13). These studies have elucidated a great deal about fetal and neonatal β cell proliferation. It is clear from the introduction that there are profound differences between mice and humans and findings in mice must be validated in humans.

Studies in humans are comparatively limited for obvious moral and ethical reasons. Developmental studies of human islets are generally restricted to histological analysis of cadaveric tissues. Nevertheless, studies in human tissues indicate the same dynamics of β cell proliferation as those seen in mice, a peak in β cell proliferation in neonatal stages and a rapid decline through adolescence, though on a time scale that reflects the difference between human and mouse life spans (7, 14). Cadaveric human pancreatic islets may be studied in culture for a relatively short time. These studies indicate that cultured neonatal islets lack the functional maturity that is typically associated with those containing proliferative β cells (15). Unfortunately, the implications of these experiments on β cell proliferation are highly correlative as it is difficult to detect β cell proliferation in cultured human islets. It is also possible to study human islets as mouse kidney xenografts, offering the islets a more dynamic, *in vivo* environment. While human β cell proliferation has been observed, and even stimulated, in these models (16), the study of neonatal islets in this context has been difficult due to a lack of availability.

### Adult β Cell Proliferation

The steep decline in β cell proliferation after the early neonatal period results in fixation of β cell mass during adolescence (7). Human β cell proliferation peaks around 4% during the early neonatal period and, under normal physiological conditions, decreases to approximately 0.2% in adults (14). In mice, the β cell proliferation rate is generally higher, reaching approximately 10% during development and resting at approximately 1% in adults (17). Unlike many other tissues in the body that have the capacity to regenerate, proliferation in adult pancreatic islets is only sufficient to maintain a relatively stable endocrine cell mass. Adult β cells lack the proliferative capacity to compensate for severe loss of β cells, as inflicted during T1D and T2D progression. There are, however, physiological circumstances under which adult β cells are stimulated to proliferate. These circumstances indicate that adult β cells maintain the capacity to proliferate at higher rates than normally seen and offer hope that stimulated β cell proliferation may be a viable option for the restoration β cell mass after diabetic loss.

Pregnancy results in an increased demand for insulin and in the mouse this is accompanied by an adaptive expansion of the β cell population (18). This proliferative response is driven by the placental lactogens and prolactin in mice (19, 20). However, the mechanism of this β cell expansion, whether due to proliferation or *de novo* differentiation from progenitors, is still in debate. Autopsy studies revealed little increase in proliferation marker Ki67, in the pancreas of pregnant women (21). While human β cell mass typically increases through hyperplasia, the lack of proliferation markers led to the interpretation that the observed increase in β cell mass was the result of β cell neogenesis through progenitor differentiation. Early *in vitro* studies indicated that placental lactogens, prolactin and human growth hormone can all stimulate β cell proliferation in cultured human islets (19). However, more recent studies have indicated that, while activation of downstream pathway components of prolactin signaling (*i.e.*, STAT5A) could upregulate prolactin signaling targets, they failed to stimulate human β cell proliferation (22). In all, the story of pregnancy-induced β cell expansion is complex and difficult to translate to humans. Many of the lessons learned from the experiments described above appear to be more relevant to studies of gestational diabetes than to generalized β cell proliferation.

Similar to pregnancy, non-diabetic obesity induces insulin resistance and a compensatory increase in β cell mass (23). Although obesity-induced T2D ultimately results in decreased β cell mass, prediabetic obesity causes an initial increase on β cell mass prior to the development of T2D (24–26). Mouse models of obesity and insulin resistance, both genetic and high fat diet, show profound increases in β cell mass (27, 28). These studies have elucidated some molecular pathways for adult β cell proliferation and indicate that intracellular glucose signaling drives β cell proliferation, during hyperglycemia, through glucose metabolism pathways (29–31). Confounding the translatability of these studies, human islets xenograft studies have demonstrated that, despite profound β cell proliferation in native mouse pancreas, human β cells from islets transplanted under the kidney capsules of the same mice did not proliferate in response to high fat diet (32). Again, the differences in β cell plasticity between humans and mice make it difficult to ascertain the mechanisms of stimulated adult β cell proliferation.

Pancreatic injury models in mice suggest that regenerative signals within the whole pancreas could stimulate β cell regeneration (6, 33, 34). However, such regeneration was observed only in young animals and it was not clear whether the β cell regeneration occurred *via* proliferation pathways or through β cell neogenesis (34). Furthermore, the issue of translatability to the human system was again raised as studies in partial pancreatectomy patients failed to demonstrate increased β cell proliferation, even after removal of up to 50% of the pancreas mass (35). Chemical injury models, such as streptozotocin treatment, have helped to elucidate some of the molecular mechanisms involved in adult β cells proliferation (36).

### Extracellular Signals Stimulating Adult β Cell Proliferation

Despite the difficulties outlined above, there has been significant progress made in understanding β cell proliferation under normal physiological conditions and some successes in regeneration of adult β cells through proliferation. Mouse studies, particularly in development, have elucidated many of the molecular components necessary for β cell proliferation. Secreted factors such as incretin hormones (37, 38), adipokines (39–42), interleukins (43, 44), liver-derived factors (45) and secreted growth factors (46–49) have been shown to stimulate β cells proliferation under dynamic physiological conditions. In addition to secreted factors, extracellular matrix components have also been shown to play a critical role in the proliferative capacity of β cells through establishing an islet microenvironment with localized growth factor signaling (50). Of particular recent interest are a family of pleiotropic matricellular proteins known as the cellular communication network (CCN) proteins. Two members of this family have been shown to stimulate adult β cell proliferation after injury and during pregnancy (CCN2/CTGF) (51) and as a secreted factor from pre-weaning mouse serum (CCN4/WISP1 (52). Recently, it has been shown that intraislet endothelial cells and macrophages contribute to β cell proliferation through VEGF signaling and remodeling of the extracellular matrix (53). These studies of extracellular stimulatory factors have paved the way for understanding intracellular signaling pathways that drive β cell proliferation and may be targeted in efforts to stimulate adult β cell proliferation.

### Intracellular Pathways Involved in β Cell Proliferation

Extracellular signals transmitted into the β cell stimulate transduction pathways that regulate cellular functions. The challenge is finding the signals that stimulate the necessary signaling pathways to stimulate β cell proliferation, so called mitogenic pathways. Much effort has been placed on elucidating mitogenic signaling pathways in β cells in order to restore a proliferative capacity to adult β cells. There are numerous mitogenic pathways found in proliferating cells throughout the body. It is important to understand the pathways that stimulate β cell proliferation in order to access a physiologically relevant mechanism for β cell regeneration. While there is a broad literature regarding many mitogenic signaling pathways, it is the pathways that have been successfully targeted to stimulate adult β cell proliferation that will be the focus here.

Cyclin dependent kinases (CDKs) drive cell cycle entry and progression and CDK inhibitors are commonly upregulated in cells that have exited regular cell cycle. This is the case in adult β cells where CDK inhibitors such as p16^INK4a^/CDKN2A, p21/CDKN1A and p18/CDKN2C are known to be key regulators of β cell proliferation (17). A major role of mitogenic pathways in β cells is to overcome the actions of these kinases and allow β cells to progress into active cells cycle.

The most successful identification of β cells mitogens has come through studies designed to identify soluble signals that stimulate β cell proliferation. Using high throughput methods, DYRK1A inhibitors were shown to stimulate adult human β cell proliferation implicating the Calcineurin/NFAT/DYRK1A pathway as a major mitogenic pathway in β cells (54). At the same time, studies demonstrated that osteoprotegerin, a target of prolactin receptor signaling, could stimulate β cell proliferation through the RANKL binding and modulation of the CREB and GSK3 pathways (36). Mouse studies identified a liver-derived mitogen, serpinB1, that stimulates β cells proliferation in zebrafish, mouse, and human adult β cells by stimulating MAP kinase pathway and inhibiting GSK3 (45). A similar effect in mouse and human adult β cells was observed with inhibition of the KRAS signaling pathway and its downstream effector RASSF1A, or by stimulating ERK1/2 phosphorylation with a GLP-1 agonist in combination with inhibition of the tumor suppressor Menin (55). Finally, studies of CDK inhibitors demonstrated that decreased expression of p18/CDKN2C and p21/CDKN1A resulted in adult human β cell proliferation (56). In all, at least four distinct mitogenic pathways and two cell cycle regulators important for adult β cell proliferation were identified during this short time. Given that mouse β cells have greater plasticity with regard to proliferative capacity, mouse studies were sufficient to identify these individual mitogenic pathways. However, none of these pathways, individually, produced enough proliferation in human β cells to be considered a reasonable treatment for diabetic β cell loss.

More success in the human setting has come from studies of multiple mitogenic pathways. Combined inhibition of the Calcineurin/NFAT/DYRK1A pathway with the DYRK1A inhibitor, Harmine, and the TGFβ signaling pathway, with inhibitors such as ALK5, resulted in a synergistic increase in β cell proliferation in primary cultures and human islet xenografts (16). The TGFβ signaling pathway is commonly known as being antagonistic to mitogenic signals in many tissues. This combinatorial approach may produce a robust enough proliferative response in β cells to be of clinical use in stimulating their regeneration.

### Proliferation of α Cells

Regeneration of β cells through stimulated proliferation has been, so far, the focus of this section. While this is not as well characterized as for β cells, all of the endocrine cells in the pancreatic islet appear to follow the same dynamic pattern of proliferation as β cells, *i.e.*, highest proliferation in the early neonatal period with a steep decline in proliferation into adulthood (14). There are well defined physiological conditions under which adult pancreatic α cells will be stimulated to proliferate. Interrupted glucagon signaling, either genetically or pharmaceutically, has been shown to result in robust α cell proliferation (57–60). Such interruption inhibited amino acid uptake in the liver resulting in higher serum amino acid levels which ultimately stimulated α cell proliferation (57). This phenomenon is well characterized and has been observed in zebrafish, mice, and human islet xenografts (57, 58, 61). While not a direct stimulation of β cell regeneration, α cells have been shown to transdifferentiate into β cells under certain conditions as discussed below. Stimulated α cell proliferation coupled with α-to-β cell transdifferentiation could be another route to β cell regeneration. This method of stimulating proliferation in cells other than β cells followed by stimulated transdifferentiation may be especially necessary in individuals with long term T1D where β cell mass may be so low that directly stimulating β cell proliferation is not a viable option.

## Endocrine Cell Transdifferentiation and Endocrine Progenitors

Diverse lines of research have demonstrated the capacity of endocrine, as well as acinar, ductal and centroacinar cells to generate new β cells *via* a range of different mechanisms. A number of studies seeking to establish conditions promoting endocrine cell interconversion or identify endocrine progenitors residing in the islet were inspired by the fact that all endocrine cells are derived from dedicated Ngn3^+^ progenitors and thus bear a close lineage relationship.

### Endocrine Transdifferentiation

Genetic studies showed that ectopic expression of *Pax4* or inactivation of *Arx* in α cells resulted in the neogenesis of functional β cells from α cells (62, 63). Similarly, inactivation of the tumor suppressor *Men1* in α cells triggered their conversion into β cells albeit with the concurrent development of insulinomas (64). The predisposition of α cells for this transdifferentiation has been attributed to the similar chromatin states of α and β cells (65). This propensity was further confirmed following extreme conditions of β cell ablation using the diphtheria toxin receptor (DTR) approach. When β cells were nearly eliminated in post-pubescent mice, transdifferentiated α cells accounted for a large proportion of newly generated β cells. This transdifferentiation capacity is absent in pre-pubescent mice, however, in these mice, δ cells can transdifferentiate into β cells in a FoxO1 dependent manner. Both processes generated bi-hormonal cells suggesting a mechanism of gradual conversion (66, 67). This conversion was accelerated in mice by the combined inactivation of *Arx* and *DNA methyltransferase 1* (*Dnmt1*). Interestingly, combined loss of these genes and expression of β cell markers were observed in α cells of T1D human islets suggesting that this transdifferentiation may actually be employed in humans for β cell mass replenishment (68). Moreover, *in vitro* re-aggregated adult human α or PP cells acquired some aspects of β cell physiology including insulin production and glucose sensing following ectopic expression of *PDX1* and *MAFA* (69).

Pharmacological stimulation of α-to-β cell conversion would provide a possible means of β cell mass restoration in diabetes. It has been suggested that artemisinins, which stimulate GABA signaling, or GABA long term administration trigger this conversion by translocating Arx to the cytoplasm and promoting its subsequent degradation (70, 71). However, these results are still debated as other studies using lineage tracing or *in vitro* culture of mouse and human islets were not successful in inducing this conversion (72, 73). Other interesting possibilities include two kinase inhibitors which target ribosomal S6 kinase (RSK) and cyclin-dependent kinase-2 (CDK2) and, at least *in vitro*, upregulate multiple β cell markers including PAX4 in a dose dependent manner (74, 75). In addition, stimulation of the liver receptor homologue-1 (LRH-1/NR5A2), a nuclear receptor that represses inflammation in digestive organs, with a small molecule agonist has been shown to increase β cell (76) mass in pre-clinical mouse models of experimental autoimmune diabetes. An increase in the number of α/β bihormonal cells and the repression of the α cell genetic program, following the administration of the agonist, suggested that the implicated mechanism was an α to β conversion, but this was not shown by lineage tracing.

The forced conversion of α to β cells resulted in a significant number of cells that persisted as bi-hormonal cells suggesting that the conversion proceeded through distinct steps rather than a sudden, all or none, transformation. This interpretation was strengthened by the recent identification of ‘virgin’ β cells that express insulin but lack other key β cell markers, do not sense glucose, and do not support calcium influx. These cells reside in a specialized niche at the periphery of the murine islet, which supports α to β cell interconversion, and correspond to an intermediate cell type between α and β cells constituting a life-long source of fully functional β cells. Interestingly, such immature cells can be identified in the normal human pancreas from donors of different ages and from T1D donors, suggesting that these cells could be a plausible candidate for the replenishment of β cell mass from endogenous sources (77).

It has been shown in rat and mouse studies that stressors such as pancreatectomy induced hyperglycemia (78), loss of *FoxO1* expression combined with multiparity or aging (79) and hyperglycemia induced by the ectopic expression of an activated KATP channel (80, 81) resulted in β cell dedifferentiation marked by loss of expression of β cell identity genes, degranulation and upregulation of disallowed genes. Glycemic normalization could either completely or partially reverse this dedifferentiation depending on its timely or delayed implementation, respectively (80–82). Such dedifferentiation has also been seen in diabetic humans and, if substantial, it would provide a possible means for β cell mass recovery. This is still a matter of debate as estimates of the presence of dedifferentiated hormone^-^/synaptophysin^+^ islet cells in diabetics vary from as little as 2% (83, 84) to as much as nearly 17% (85). The number of these cells may depend on the time elapsed from the onset of the disease and its severity. Their restoration could be combined with α cell transdifferentiation. A recent study showed that this is precisely what happened when autoimmunity of NOD mice was reversed, and animals were treated with a combination of gastrin and EGF (86).

### Endocrine Progentiors

Resident progenitor cells in the islet could provide yet another route for β cell mass restoration. Such a dedicated population, at least in the mouse, appears to be ghrelin producing cells (ϵ cells). They represent 5-10% of the total endocrine cells at birth but their numbers decline significantly postnatally (87, 88). Lineage tracing studies showed that ϵ cells contribute half the population of PP cells, approximately 25% of α cells as well as rare β cells in the islets of adult mice. Intriguingly, the generation of a subset of these cells is Ngn3 independent (89). A fasting mimicking diet in mice could induced the stepwise expression of Sox17 and Pdx1 leading to Ngn3-driven generation of β cells. Repeated cycles of this diet restored normoglycemia in mouse models of T1D and T2D. This regime had similar effects in T1D human pancreatic islets, and those effects could be recapitulated by PKA and mTOR inhibition (90). Whereas the authors suggested that these treatments drove reprogramming of endocrine cells, the possibility of a mobilization of resident endocrine progenitors has not been addressed. Intriguingly, persistent, functionally important expression of Ngn3 at both the mRNA and protein levels has been detected in mouse islets (91) providing a possible mechanistic underpinning for the generation of new β cells from resident islet cells. Importantly, a recent study identified a small cell population in the mouse islets comprising just 1% of the endocrine cells. These cells have a distinct transcriptional signature that is highly reminiscent of Ngn3^+^ embryonic endocrine progenitors and are lacking markers of terminally differentiated pancreatic cells. They are marked by strong expression of *Procr*, a cell surface marker enriched in stem cells of other organs. Procr^+^ cells originate from Ngn3^+^ cells and clonal analysis *in vivo* showed that they differentiate into all types of endocrine cells while self-renewing. Furthermore, these could support organoid growth *in vitro* in the presence of endothelial cells and differentiated into functional β cells (92). The identification of a similar human population would certainly open additional exciting prospects.

## Acinar Cell Transdifferentiation

As a candidate source cell population for diabetes cell therapy, acinar cells have the obvious advantages of abundance and demonstrated proliferative capacity and plasticity. In humans, acinar cells constitute more than 50% of the mass of the organ and an abundance of acinar cells are left over from islet isolation procedures. The acinar compartment contains actively proliferating subpopulations such as Bmi1^+^ cells as well as quiescent progenitors such as Dclk1^+^ and Stmn^+^ cells that can mediate complete acinar restoration following cerulein mediated acute pancreatitis (93–95). Acinar cells can undergo transdifferentiation into progenitor, duct-like cells during a process known as acinar to ductal metaplasia (ADM) that facilitates regeneration after injury. This process can also be initiated by oncogenic signalling in which case the transdifferentiated cells become the precursors of pancreatic ductal adenocarcinoma (PDAC) (96). On the other hand, none of these actively proliferating or quiescent acinar subpopulations has demonstrated a capacity for β cell differentiation and ADM does not entail the generation of Ngn3^+^ endocrine progenitors, let alone β cell neogenesis. Additionally, acinar cells harbour extensive repressive histone modifications of endocrine genes that need to be removed before endocrine conversion (65).

The abundance of acinar cells inspired a number of studies where acinar cells were cultured in suspension under different conditions with the aim to generate functional β cells. Such cultures of rat acinar cells resulted in extensive cell death and transdifferentiation to mostly duct cells but addition of EGF and LIF gave rise to a number of endocrine cells which was increased by additional Notch inhibition. The cells were shown to be of acinar origin by acinar cell specific incorporation of lectin and they were able to rescue diabetes in alloxan-treated mice (97, 98). In a similar approach, suspension culture of mouse acinar cells in the presence of EGF and nicotinamide resulted in β cells comprising 5% of the cultured cells and lineage tracing suggested that these were indeed derived from elastase/amylase expressing cells (99). Such encouraging findings were not restricted to rodents since non-endocrine adult human pancreatic epithelial cells supported endocrine differentiation after *in vitro* expansion and subsequent transplantation under the mouse kidney capsule (100). In this study, the origin of the cells was not lineage traced but a later, similar, study showed that half of the elastase 2A promoter lineage traced human acinar cells, transduced with STAT3 and activated MAPK, upregulated Ngn3 expression and that 3D culture promoted their conversion to β cells (101).

Acinar cell conversion into β cells has not been detected in the uninjured mouse pancreas but temporal labelling of acinar cells using a *Ptf1a^CreERT2^* driver and pancreatic duct ligation showed a small, long term contribution of acinar cells to endocrine cell generation *via* a duct-like intermediate. The conversion was enhanced when the number of pre-existing β cells was reduced by streptozotocin treatment (102).

Although not directly addressed in the studies mentioned above, *in vitro* conversion following relatively long periods of cell culture or conversion *in vivo* following injury imply that the underlying mechanism would be dedifferentiation of a small number of competent acinar cells, possibly followed by proliferation, and final endocrine differentiation. However, acinar cells also have the capacity for transdifferentiation following transduction by systemically administered viruses that expressed a cocktail of the Pdx1, Ngn3 and MafA transcription factors. Subsequent studies showed that transgenic expression of the same combination of transcription factors or even just Pdx1 through the elastase 2A promoter could induce the transdifferentiation of a few acinar cells into endocrine cells including β cells (103–105). This transdifferentiation appears to be inhibited under hyperglycemic conditions (106).

A common thread in all these studies is that only a small number of acinar cells can undergo transdifferentiation. Single cell RNA Seq analyses may provide the keys to identify these elusive cells and the molecular pathways involved in their transdifferentiation.

## Duct Cell Transdifferentiation

The ducts have been postulated as a source of islet cells since the beginning of the 20^th^ century (107, 108). The image of an islet emerging from an adult human duct published in the 1980s (109), the subsequent observations that rodent and human islets or single endocrine cells are very often associated with ducts in adults (110, 111) and the common developmental lineage of duct and endocrine cells strengthened the interest in this hypothesis.

This hypothesis was initially pursued in rat and mouse injury models. Rat pancreatectomy (Px) resulted in duct dedifferentiation and expansion followed by recapitulation of aspects of embryonic development to form apparently new pancreatic lobes containing islets (112). Regeneration of the β cell mass following rat pancreatectomy was enhanced by long-term administration of gastrin (113). Px experiments in mouse lead to contradictory results with some groups reporting both β cell neogenesis and replication of pre-existing β cells (114, 115) whereas others detected only enhanced β cell replication (6, 116). Pancreatic duct ligation (PDL) resulted in the appearance of small islets or isolated Ins^+^ cells near the ducts as well as in the appearance of Nng3^+^ cells. The latter were able to give rise to endocrine cells *in vitro* (117, 118). However, these PDL studies raised controversy due to reproducibility issues and it was also suggested that changes in the composition of the tissue due to the surgical procedure might have led to misinterpretation of the results (119, 120). In any case, these procedures trigger an immune response and cytokines have been shown to stimulate duct proliferation, epithelial to mesenchymal transition and the initiation of the endocrine differentiation program through the upregulation of Ngn3 (121–123). Extensive acinar and endocrine ablation using the DTR system (124) led to duct cell driven regeneration of endocrine cells. This approach induced less inflammation and may therefore have fewer confounding effects (125). Apart from the controversy that some of these experiments generated, none of these approaches included rigorous lineage tracing to rigorously demonstrate the ductal origin of the additional β cells.

This has been attempted in other studies using a large number of diverse Cre driver lines. An early study, using a 1.6 Kb piece of the exclusive duct marker carbonic anhydrase II (*CAII*) promoter to drive Cre expression, suggested that duct cells contributed to β cell neogenesis, particularly following PDL (126). However, the fidelity of this transgene was questioned and an *in vitro* study using lineage tracing and human duct cells concluded that it was CAII^-^ but not CAII^+^ cells that could be converted into β cells (127). Two studies using BAC transgenic lines expressing the inducible form of Cre from the full set of *Hnf1b* or *Sox9* regulatory elements found that, whereas Hnf1b^+^ and Sox9^+^ cells generate endocrine cells during development, they stop doing so after birth (128, 129). In contrast, a study using a different *Sox9* BAC transgenic line suggested that Sox9^+^ cells can generate β cells in the context of mild hyperglycemia and long-term administration of EGF and gastrin (130). On the other hand, knock-in approaches using the duct specific *Krt19* and *Muc1* loci to drive expression of *CreERT* failed to detect a contribution of duct cells to β cell neogenesis.

Other studies using genetic manipulations combined with lineage tracing demonstrated the ability of some duct cells for β cell neogenesis. Ectopic expression of *Pax4* in α cells rescues toxin induced diabetes by converting α to β cells and the former were replenished from Ngn3^+^ cells of ductal origin (131). In adult, TGF-β receptor mouse mutants, β cells arose from intra-islet ducts following partial pancreatectomy (132) suggesting that suppression of TGFβ signalling may enhance duct cell conversion into β cells. Deletion of the tumor suppressor ubiquitin ligase *Fbw7* specifically in duct cells led to the upregulation of *Ngn3* expression in a small subset of duct cells and their subsequent conversion into functional β cells (133).

Taken together, and despite reservations for some of the experimental approaches and/or their interpretations, these findings suggested that some duct cells may have the capacity to contribute to β cell neogenesis. A number of *in vitro* and transplantation studies of both mouse and human duct cells have strengthened this notion.

Human duct cells present in islets proliferated and induced *Nkx6.1* expression following transplantation under the kidney capsule provided that the mice carried growth stimulatory traits (134). This was supported by pharmacological treatments using BMP7 (127), a combination of EGF and gastrin (135) or preadipocyte factor 1 (136). These approaches suggested that rodent and human duct cells could proliferate and differentiate into endocrine cells *in vitro* and they were also used to reverse diabetes in mice. Expansion of human ductal tissue in matrigel resulted in the formation of cysts that contained functional islet-like structures (137). However, the possibility that the initial tissue contained contaminating islet cells was not excluded and a similar approach later showed that removal of endocrine cells by NCAM-mediated magnetic sorting severely depleted the endocrine differentiation potential of these cells (138). To reduce or eliminate contaminating endocrine cells several presumed duct specific markers have been used.

Human duct cells isolated by FACS using the carbohydrate antigen 19-9 expanded *in vitro* and, when transplanted into NOD-SCID mice, 1% of them were converted into insulin producing cells (139). This suggested that a small percentage of duct cells may retain progenitor properties, and this has been confirmed by several studies. Indeed, only a subset of duct cells has organoid forming capacity. For example, CD24^+^ duct cells isolated from mouse expand as spheroids and show endocrine differentiation potential (140). CD133 is another duct progenitor marker in both humans and mice. Mouse CD133^high^/CD71^low^ mouse cells are enriched in tripotent colony forming progenitor cells (141). Human duct cells expanded *in vitro* showed increased expression of NGN3 with CD133 co-expression and FACS isolated CD133^+^ cells from these cultures showed endocrine differentiation potential (142). Finally, expression of the key endocrine developmental regulators NGN3, MAFA, PDX1 and PAX6 in expanded CD133 human cells converted duct cells into endocrine cells with β-cell properties. Tripotent progenitor cells have been identified in the human main ducts as P2RY1^+^/ALK3^+^ cells that can differentiate into all pancreatic lineages upon transplantation, including functional β cells. Consistent with that, single cell RNA Seq analyses suggested the presence of three distinct differentiation axes (143).

In assessing all the results presented above for their potential in diabetes therapy it is important to bear in mind that the ductal tree is an intricate network containing intercalated ducts, intra-and interlobular ducts as well as main ducts. Molecular analyses at the single cell level identified at least four different duct cell types (144) and different experiments may mobilize different subsets of duct progenitors or induce dedifferentiation of different subsets of duct cells. The challenge will be to identify the cells of origin with unique molecular markers and the distinct signals implicated in their mobilization.

## Centroacinar Cells as Progenitor Cells

Pancreatic centroacinar cells (CACs) exist in all vertebrates. CACs are rare, specialized cells situated in the middle of the acinar lobules. They are small, with minimal cytoplasm, numerous mitochondria, and long cytoplasmic extensions, some of which reach the endocrine compartment. They are contiguous with ductal cells and form a fenestrated lining on the luminal surface of each acinus (145, 146). They were first identified in the rodent pancreas in the early 1960s and it has been suggested that they may participate in pancreas regeneration following ethionine mediated ablation of acinar cells (147, 148). It has been postulated that CACs originate developmentally from a domain at the interface of bipotent trunk cells and tip acinar progenitors thus possibly retaining their progenitor status (119, 145). Their expression profile supports this hypothesis as CACs are Notch responsive (149) express Hes1 and Sox9 together with Ptf1a (150) and proliferate in response to injuries such as streptozotocin-induced destruction of β cells, partial pancreatectomy and cerulein induced acute or chronic pancreatitis (151–153). Lineage tracing of Notch responsive cells in the adult zebrafish following β cell ablation or partial pancreatectomy showed that they give rise to endocrine cells. However, lineage tracing in the adult mouse using either a replacement knock-in *Hes1^CreERT2^* allele or a BAC *Sox9^CreERT2^* allele did not detect a contribution of labelled cells in β cell neogenesis in either the resting pancreas or following PDL (128, 154). The latter experiments relied on the assumption that all CACs expressed either of the two markers but given the scarcity of these cells and their small size this was far from certain. The lack of a unique marker for CACs has hampered progress but the finding that mouse CACs can be distinguished and isolated by virtue of their high aldehyde dehydrogenase (ALDH) activity provided plausible candidates among the several *Aldh* isoforms. Moreover, ALDH^high^ cells differentiate into endocrine or acinar cells *in vitro* either forming pancreatospheres in suspension culture or when injected in embryonic pancreatic buds (150). Aldh1b1, an Aldh isoform, is expressed in all mouse pancreas progenitors during development and its expression is retained specifically in CACs in the adult (155). Lineage tracing using a knock-in *Aldh1b1^CreERT2^* allele that retained all the regulatory elements and the functionality of the targeted *Aldh1b1* allele suggested that Aldh1b1 expressing cells are self-renewing and contribute to all three pancreatic lineages under homeostatic conditions. Moreover, mouse Aldh1b1 expressing cells were necessary and sufficient to form pancreatic organoids *in vitro* (156). Similarly, only ALDH^high^ cells from adult human pancreas can form organoids and show endocrine differentiation potential *in vitro* and *in vivo* after transplantation (157). Immunofluorescence experiments suggested heterogeneity of the Aldh1b1 expressing mouse CACs and single cell RNA Seq showed that these cells are not all identical but are distributed along two main paths of differentiation and that the earliest progenitors among them are devoid of detectable *Sox9* and *Hes1* expression (156).

Identification of additional surface markers and elucidation of self-renewing and differentiation requirements may provide the means to isolate, expand and differentiate human CACs into endocrine cells for diabetes treatment.

## Discussion - Pros and Cons Towards Clinical β Cell Regeneration

Single cell RNA Seq has revealed that there is significant heterogeneity among pancreas differentiated cells that were once considered homogeneous stable populations (143, 144, 158–160). Terminally differentiated, mitotically inactive cells may exist in a dynamic equilibrium with proliferating differentiated cells and progenitors. This might be the underlying reason for the bewildering variety of pancreatic responses to injury and disease. At the same time, it may provide the opportunity for multiple points of intervention to restore β cells mass.

**Figure 1:**
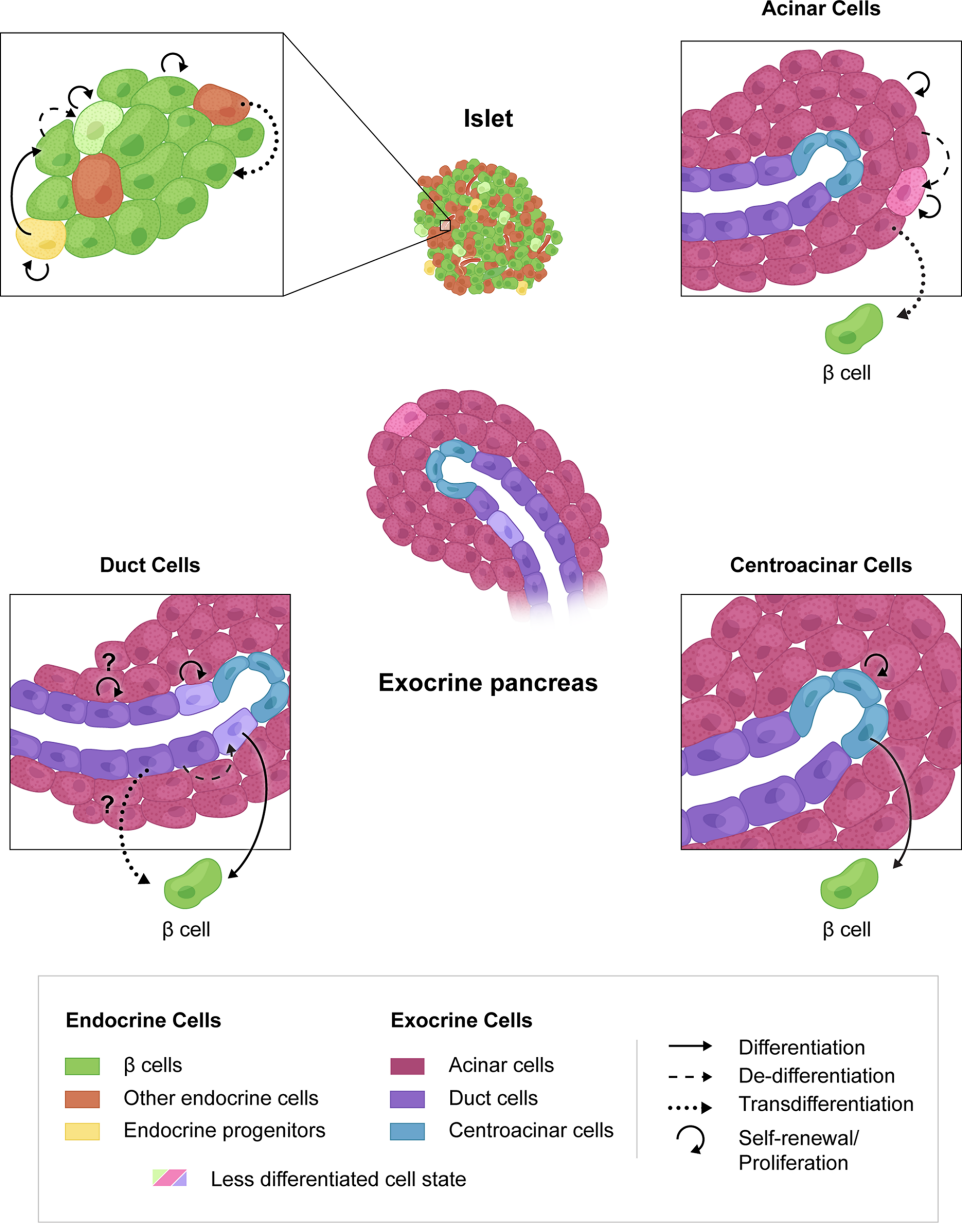
Mechanisms of β cell regeneration through proliferation, progenitor differentiation or transdifferentiation. β cell proliferation is the most direct route to regeneration of β cell mass (upper left, circular arrow). The presence of endocrine progenitors in human islets is unknown but could lend to increased β cell mass. Transdifferentiation from other endocrine cells (upper left), acinar cells (upper right), duct cells (lower left) or centroacinar cells (lower right) may also be targeted for β cell regeneration. Possible cell interconversions that have not been shown experimentally are indicated by question marks.

Accordingly, there are multiple possible approaches to β cell replenishment that have been mainly explored in mice but show some promise of applicability to humans (**Figure 1**). The most direct route for restoration of β cell mass would stimulate the proliferation of existing β cells. This is happening *in vivo*, particularly in rodents, even under physiological conditions and several mitogenic pathways have been identified that show promise when manipulated in combination (16, 54, 161, 162). Transferring these to a clinical setting will not be without challenges since the proliferation rate of human β cells is much lower than that in rodents. Additionally, in severe diabetes one will have to rely on a greatly diminished mass of stressed β cells (**Table 1**).

Dedifferentiated β cells are another likely source for β cell replenishment but the extent of their presence in the diabetic pancreas is controversial (83, 84). It may depend on the stage of the disease as it is possible that advanced, long term diabetes may exhaust this cellular pool. The upregulation of progenitor markers in these cells suggests that they would be more amenable to stimulation of proliferation. This could be combined with treatments that promote β cell differentiation as a consequence of autoimmunity reversal and restoration of normoglycemia (86). Such mobilization of dedifferentiated β cells could be combined with α cell proliferation and transdifferentiation (77) (**Table 1**).

Progenitor cells are expected to be more proliferative, while remaining malleable towards β cell differentiation. The identification of dedicated progenitor cells in the murine islet (89) has generated excitement but, so far, remains an exclusive murine feature. Murine centroacinar cells, or at least a subpopulation thereof, express progenitor markers, remain proliferative, can contribute to all three pancreatic lineages under homeostatic conditions (89) but it remains unknown if they can be stimulated to generate β cells under diabetic conditions. A possible path would be their isolation, *in vitro* expansion, and differentiation into β cells followed by transplantation. However, specific surface markers that would mediate their efficient and specific isolation have not been identified (**Table 1**).

Transdifferentiation is another major route that may be useful in restoring β cell mass. Because of greater similarity and physical proximity, α cells would be the cell of choice and their transdifferentiation has been well documented by lineage tracing in murine models. Additionally, they are more proliferative than β cells, have defined proliferation pathways and their proliferation is further stimulated by β cell destruction (57–60, 67). However, because of the presence of bi-hormonal and virgin β cells, it is questionable whether this differentiation is sufficiently driven to completion, and it remains unclear whether it can occur in human islets. Acinar cells are abundant, contain proliferative subpopulations and it has been established that a small subpopulation is amenable to β cell conversion *via* transcription factor mediated reprogramming (103–105). However, their tendency is to rather differentiate into duct cells and transdifferentiation into β cells in either the normal or injured pancreas has not been documented. Duct cells as a possible β cell source have remained controversial. Ducts contain diverse subpopulations that can be mobilized using different stimuli and rodent injury models have repeatedly suggested their transdifferentiation capacity which could be recapitulated *in vitro* (139). However, β cell proliferation has been proposed as the reason for apparent β cell neogenesis and convincing lineage tracing experiments are scarce with the exception of a model where the tumor suppressor gene *Fbw7* has been inactivated in duct cells (139). Evidence that this transdifferentiation can happen *in vivo* is lacking and stimulation of duct regeneration by cytokines may simultaneously adversely affect β cell function.

Irrespective of the relative merits of mechanisms and possible cell sources, there are general concerns to be addressed. Two general routes appear possible for the restoration of β cell mass. The first would be *in vivo* stimulation of proliferation, differentiation, transdifferentiation or a combination thereof. This would involve delivery of small molecules or transcription factors to the target population and specificity of the delivery would be of major importance to ensure both efficiency and elimination of side effects. There are several promising avenues for targeting β cells (163–166) but knowledge of the receptors present on the surface of other target cells will be essential. This necessitates the unequivocal identification and thorough characterization of the target cells, but this remains elusive even in mouse models. An additional risk to be evaluated is the possibility that stimulation of endocrine cell proliferation may result in the development of insulinomas or glucagonomas. In this respect transdifferentiation may appear a safer, albeit slower, route. The other route would be the isolation of progenitor cells from a biopsy followed by *in vitro* expansion and differentiation to provide enough β cells for transplantation. This remains a daunting undertaking as it requires the identification of specific surface molecules, to enable efficient isolation, as well as the growth requirements for expansion *in vitro*. Additionally, transplantation of the differentiated cells will face the same hurdles as hPS cell derived endocrine cells regarding the suitability of candidate transplantation sites, adequate oxygenation, the use of macroencapsulation devices to protect against the escape of undifferentiated cells and the development of fibrotic tissue around the device that would eventually block the function of the transplanted cells.

Beyond these challenges, choosing between the method of β cell regeneration is confounded by the difficulty in evaluating β cell mass in individuals to be treated. As mentioned above, both T1D and T2D are associated with loss of β cell mass with time. The difficulty lies in the heterogeneity of β cell loss among individuals, even those with the same duration of disease. In some individuals with T1D, there may be sufficient residual β cell mass to consider treatment that will stimulate proliferation whereas in other individuals, the loss of β cells may be so severe that a neogenic treatment may be required. Further compounding this problem is a difficulty in assessing β cell mass. Current technologies allow β cell mass calculation in cadaveric pancreas samples, but we lack the imaging technology to assess β cell mass in living patients. This further highlights the challenges to the development of β cell regenerating therapies.

In summary, whereas there are several promising possibilities to restore β cells using adult pancreatic cells significant challenges remain and require additional research.

## Data Availability Statement

The original contributions presented in the study are included in the article/supplementary material. Further inquiries can be directed to the corresponding authors.

## Author Contributions

All authors reviewed and edited the final manuscript. All authors contributed to the article and approved the submitted version.

## Funding

Work in the authors’ laboratories was supported by funding provided by the National Institute of Diabetes and Digestive and Kidney Diseases [the Human Islet Research Network (HIRN; RRID : SCR_014393; https://hirnetwork.org; DK112232, DK123716, DK123743, DK104211, DK108120), and by DK106755, DK117147, DK20593] (AP), The Leona M. and Harry B. Helmsley Charitable Trust, and the Department of Veterans Affairs (BX000666)(AP), the German Center for Diabetes Research (DZD)(82DZD00101) (AG) the German Research Foundation (DFG)(GA 2004/3-2 and IRTG 2251)(AG), and the Greek General Secreteriat of Research and Technology (GSRT) (Code: T1EDK-03532)(IS).

## Conflict of Interest

The authors declare that the research was conducted in the absence of any commercial or financial relationships that could be construed as a potential conflict of interest.

## Publisher’s Note

All claims expressed in this article are solely those of the authors and do not necessarily represent those of their affiliated organizations, or those of the publisher, the editors and the reviewers. Any product that may be evaluated in this article, or claim that may be made by its manufacturer, is not guaranteed or endorsed by the publisher.
